# Rural South African Community Perceptions of Antibiotic Access and Use: Qualitative Evidence from a Health and Demographic Surveillance System Site

**DOI:** 10.4269/ajtmh.18-0171

**Published:** 2019-04-15

**Authors:** Jocelyn Anstey Watkins, Fezile Wagner, Francesc Xavier Gómez-Olivé, Heiman Wertheim, Osman Sankoh, John Kinsman

**Affiliations:** 1 Division of Health Sciences, Warwick Medical School, The University of Warwick, Coventry, United Kingdom;; 2 Medical Research Council, Wits Rural Public Health and Health Transitions Research Unit (Agincourt), School of Public Health, Faculty of Health Sciences, University of the Witwatersrand, Johannesburg, South Africa;; 3 Wellcome Trust Major Overseas Programme, Oxford University Clinical Research Unit, Hanoi, Vietnam;; 4 Nuffield Department of Medicine, Centre for Tropical Medicine, University of Oxford, Oxford, United Kingdom;; 5 Department of Medical Microbiology, Radboudumc Center for Infectious Diseases, Nijmegen, The Netherlands;; 6 Faculty of Health Sciences, School of Public Health, University of the Witwatersrand, Johannesburg, South Africa;; 7 International Network for the Demographic Evaluation of Populations and their Health (INDEPTH) Network, Accra, Ghana;; 8 Statistics Sierra Leone, Freetown, Sierra Leone;; 9 Faculty of Medicine, Department of Public Health and Clinical Medicine, Epidemiology and Global Health (Umeå Centre for Global Health Research), Umeå University, Umeå, Sweden;; 10 Department of Public Health Sciences, Global Health (Division of International Health - IHCAR), Karolinska Institutet, Stockholm, Sweden

## Abstract

Knowledge and practices of rural South African populations with regard to antibiotic access and use (ABACUS) remain understudied. By using the case of four villages in the north east of the country, our aim was to investigate popular notions and social practices related to antibiotics to inform patient-level social interventions for appropriate antibiotic use. To achieve this, we investigated where community members (village residents) were accessing and sourcing medication, and what they understood antibiotics and antibiotic resistance (ABR) to be. Embedded within the multicountry ABACUS project, this qualitative study uses interviews and focus group discussions. A sample of 60 community members was recruited from the Agincourt Health and Demographic Surveillance System, situated in Mpumalanga Province, from April to August, 2017. We used the five abilities of seek, reach, pay, perceive, and engage in access to healthcare as proposed by Levesque’s “Access to Healthcare” framework. Respondents reported accessing antibiotics prescribed from legal sources: by nurses at the government primary healthcare clinics or by private doctors dispensed by private pharmacists. No account of the illegal purchasing of antibiotics was described. There was a mix of people who finished their prescription according to the instructions and those who did not. Some people kept antibiotics for future episodes of infection. The concept of “ABR” was understood by some community members when translated into related Xitsonga words because of knowledge tuberculosis and HIV/AIDS treatment regimens. Our findings indicate that regulation around the sale of antibiotics is enforced. Safer use of antibiotics and why resistance is necessary to understand need to be instilled. Therefore, context-specific educational campaigns, drawing on people’s understandings of antibiotics and informed by the experiences of other diseases, may be an important and deployable means of promoting the safe use of antibiotics.

## INTRODUCTION

### The problem of antibiotic resistance (ABR).

Antibiotic effectiveness has decreased in recent years because of overuse and misuse, resulting in increasing bacterial resistance and threatening modern medicine.^1^ Antibiotic use relates to “ABR” and is a subset of “antimicrobial resistance” (AMR). Both are widely acknowledged as a public health concern and global problem.^2^ Drug-resistant infectious “superbugs” are increasing,^3–5^ and global efforts to curtail the emergence of resistance are gaining priority.^6^ In low- and middle-income countries (LMICs), the burden of infectious diseases is exacerbated by limited access to, and availability and affordability of antibiotics to treat infections.^7^ Little is known about the prevalence of ABR in sub-Saharan Africa (SAA), despite its known risks.^8^ There have been calls for continent-wide surveillance to inform empirically based treatment guidelines.^9^ The WHO has designed a “Policy Package to Combat AMR”^10^ for governments to commit to a national plan; yet to date, few African countries have initiated it.

The Center for Disease Dynamics, Economics, and Policy offers an online “Resistance Map”^11^ with search tool by country for ABR by either antibiotic or pathogen. General developments in the rise of AMR pathogens in Africa were found for commonly prescribed antibiotics in a systematic review by Tadesse et al.^2^; nonetheless, some country-level data were unavailable. The authors called for researchers to investigate the status of AMR and to identify knowledge gaps to design suitable local and global interventions. Research into access and use of antibiotics are presently limited in most SAA countries. South Africa has a relatively well-functioning health system with national surveillance systems routinely generating representative and robust data on antimicrobial use in tertiary care^12^ but not in primary care, where most citizens access antibiotics from.

### Social sciences literature on AMR and ABR.

The importance of social science research for tackling AMR and ABR is gaining momentum, with multidisciplinary projects being funded. Chandler et al.^13^ take an anthropological lens, considering ABR as “a discourse, social practice and natural fact” by describing varying works on the use of medicines by consumers and prescribers, and meanings of medicines for patients and providers. Likewise, Wood^14^ draws on sociological perspectives, arguing that the responsibility of using antibiotics responsibly is a societal problem, not only the “responsibility of government health services.”

Several authors summarize key social factors involved in antibiotic use by “agents”: providers, dispensers, and the general population, especially within primary research examples in developing countries.^15^ For example, Broom’s et al.^16^ study argues that antibiotic misuse by doctors is “better understood in terms of social relations.” Professional etiquette as a logic of practice can be resistant to change. “Antibiotics are often perceived as strong medicine, capable of curing almost any kind of disease,”^15^ with color, taste, and size as factors in determining perceived efficacy by the consumer.^17^

van der Geest^18,19^ discussed factors relating to the illegal distribution of biomedical medicine, particularly in Africa. They describe why lay people self-medicate and buy from drug peddlers due to long queues at overcrowded hospitals, not wanting others to know of their illness and weak drug control. In another article, they^20^ refer to biomedical pharmaceuticals as the “charm of medicines,” “meaningful,”^21^ and tangible, and as “time-saving commodities”^22^ with implications for social relations. Over the decades, knowledge gained from these studies around social factors of medicine prescribing and consumption lay the backdrop for our study and situate this work’s qualitative contribution.

### South African health system context and ABR policy.

South Africa’s pluralistic healthcare system is based on both traditional and biomedical healthcare.^23^ The public health system offers free primary healthcare to all citizens and hospital exemptions for those who qualify,^24^ resulting in high use of government facilities.^25^ In Gauteng Province, 96 percent of people use these.^26^ Research has documented patient reports of clinical neglect, and verbal and physical abuse by nurses, which is often based on organizational issues and professional insecurities.^27^ The government is investing in its “Community Health Worker” program to support household healthcare.^28^

The National Medicines Policy and Essential Medicines list^29,30^ underpin healthcare services. The Global Antibiotic Resistance Partnership^7,31^ aims to contribute to investigating strategies and solutions to curb AMR. In 2016, the South African National Department of Health^32^ released “AMR National Strategy Framework” to facilitate its implementation at all levels of the healthcare system. The policy documents the country’s national response plan to move beyond tuberculosis (TB) resistance, HIV/AIDS, and malaria by also focusing on acute bacterial infections.^33^

### The study and field site context.

This article focuses on an area in rural South Africa. We report on qualitative research centered around access to and use of antibiotics from a sample of people residing in the Agincourt Health and Demographic Surveillance System (HDSS) site in Mpumalanga Province. This study is embedded within the antibiotic access and use (ABACUS)^34^ project conducted in six countries in Africa and Asia. The overall project’s main purpose is to compare antibiotic access and consumption practices across communities (defined here as the population living within the catchment area of the Agincourt HDSS). This is to inform the design of and identify targets for intervention strategies that may be used to promote safe and appropriate antibiotic use.

Our study’s aim was to explore factors and practices around access and use of antibiotics and comprehension of “ABR.” By asking community members who reside in villages within the Agincourt HDSS about their experiences and perspectives, we can understand the cultural and social dimensions of their medicine use. Our specific objectives were to

• investigate where community members are accessing and sourcing healthcare treatment and antibiotics
• explore their understandings and experiences of antibiotics and ABR
• provide a context for developing social interventions for safe antibiotic use.

In this article, the definition describing the “appropriate or inappropriate” use of antibiotics is based on the biomedical view: inappropriate use is likely to make the medicine less effective in terms of treating the disease in question, whereas appropriate use will likely make it more effective and safe. What is “appropriate” from a biomedical perspective need not be appropriate from a sociocultural or from the patient’s perspective.

### Access to healthcare framework.

Sen’s Capability Approach^35,36^ was used for the design of the ABACUS project. Yet, for this study, we decided to use another framework that directly focused on access to healthcare than on capability and freedom.

**Figure 1.**
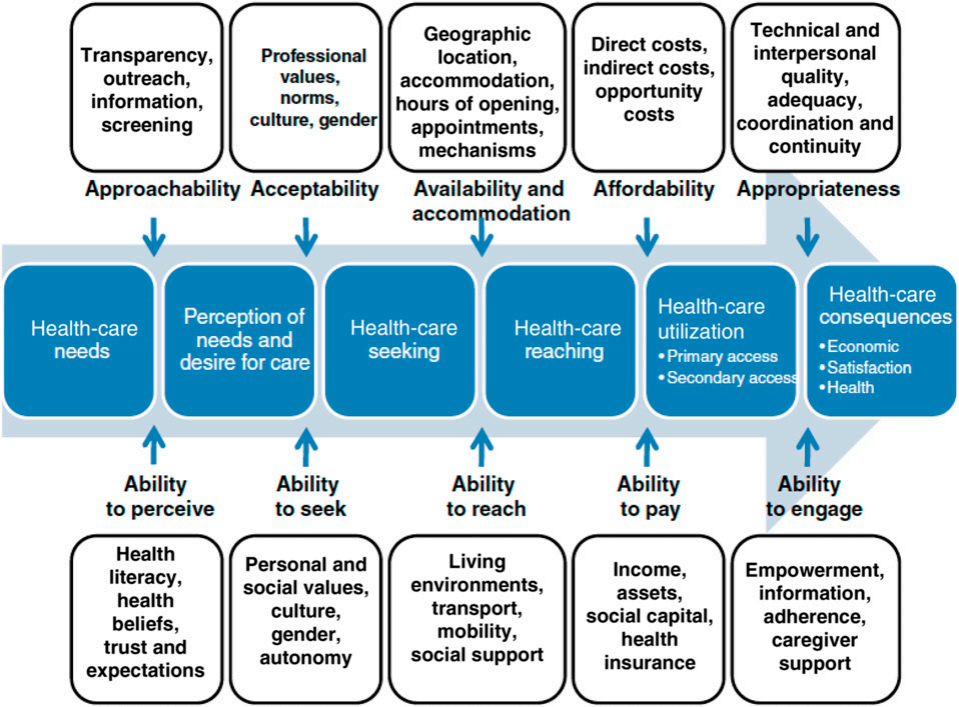
A conceptual framework of access to healthcare by Levesque et al.^37^ indicating the five dimensions of accessibility of services and five related abilities (permission to use figure granted by the publisher). This figure appears in color at www.ajtmh.org.

Access to healthcare has been described as “the opportunity to reach and obtain appropriate healthcare services in situations of perceived need for care.”^37^ Many academics have defined access to healthcare^38–40^ with most conceptual frameworks including the three dimensions of acceptability, availability, and affordability. There are varying access to healthcare models, and in this article, we use an analytical framework by Levesque et al.^37^ called “Access to Healthcare” where five abilities are discussed under the five related dimensions of accessibility. We justify this choice, given that their interpretation of access uses a patient-centered approach by conceptualizing access at the interface of health systems and populations. Also, this framework includes two further dimensions of approachability and appropriateness. Levesque et al.^37^ link each of the five dimensions to five abilities of perceive, seek, reach, pay, and engage, as illustrated in their diagram (see Figure 1). We used this analytical framework to interpret and analyze our findings, to describe the phenomena of interest, and to structure the results. We focus all five abilities of people in the process of seeking healthcare which goes beyond other available frameworks that only describe the dimensions. The various dimensions of access are not completely independent constructs and can influence each other during an episode of illness and care.

## METHODS

The research design is cross-sectional and exploratory and is used to assess and compare community-based antibiotic access and consumption, as well as the factors underpinning them. This study, is a subset of the ABACUS^34^ project under the International Network for the Demographic Evaluation of Populations and their Health Network,^41^ conducted in seven HDSS within six LMICs: South Africa, Mozambique, and Ghana in Africa; Vietnam, Bangladesh, and Thailand in Asia, further explained in Wertheim et al.^34^

### Study setting.

**Figure 2.**
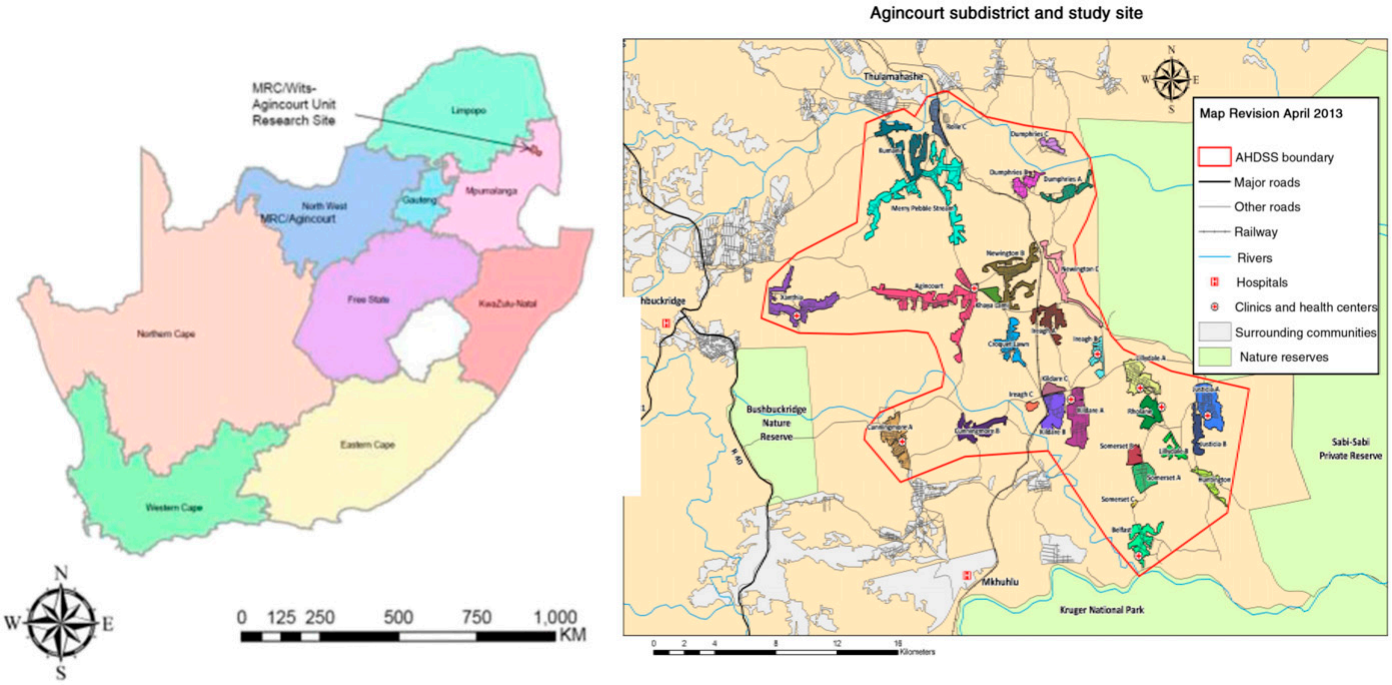
Map of the Agincourt Health and Demographic Surveillance Systems field site and its geographic location within Mpumalanga, South Africa. This figure appears in color at www.ajtmh.org.

Our article is based on data from the Rural Public Health and Health Transitions Research Unit of the South African Medical Research Council (MRC) and the University of the Witwatersrand (the MRC/Wits Agincourt Unit, referred to here as the “Agincourt HDSS”). It is located in the Agincourt subdistrict, Ehlanzeni District of Mpumalanga Province, 500 km from the city of Johannesburg (Figure 2)^42,43^ in north east South Africa.

The site was established in 1992 to support district health system development and now investigations into causes and consequences of complex health, population, and social transitions are at its core, such as observational studies and trials around preventing HIV transmission and reducing metabolic disease risk.^43^ The site covers 420 km^2^, encompassing 32 villages with approximately 16,000 households which have been under an annual population census monitoring births, deaths, and migrations to update resident status and vital events since 1992.^44–46^ These data are held in the “Agincourt HDSS database,”^47^ which we used as a sampling frame. The relational database represents the life histories of the local population (individuals and households) and takes account of in- and out-migrations.^43^ The Agincourt HDSS’s “Public Engagement” has established a long-standing relationship with the HDSS population and their leaders, based on mutual trust and respect,^46^ and it serves as a platform for information sharing between them and the community members.

The Agincourt HDSS is a typical marginalized rural community in South Africa. Unemployment rates remain high with common labor-related out-migration.^43^ Many villages are densely settled, remote, and underserved by government service providers. The district healthcare system offers citizens free primary healthcare at the point of care accessed at clinics, community health centers, and district hospitals.^48^

### Accessing the community: recruiting for community member in-depth interviews.

Participant recruitment followed a standardized approach in all ABACUS project country sites. A sample of 17 community members was randomly selected from the Agincourt HDSS database for participation in a semi-structured in-depth interview. The sample was stratified by gender and age (Table 1), with mothers with children aged five years and younger as a key population group. The remaining participants consisted of men and women who cared for children older than 5 years. Children younger than 5 years and adults older than 60 years may have a higher frequency of healthcare seeking. Household female members, particularly mothers, play a crucial role in managing childhood and family illness. Our sample was standardized and stratified to make the qualitative findings comparable from the other seven ABACUS sites in six countries and so there would be no systematic sampling bias in any given site.

**Table 1:** Respondent categories for the antibiotic access and use project Agincourt Health and Demographic Surveillance Systems in-depth interviews

Respondent categories	Female respondents	Male respondents	Total
Mothers who care for children aged 5 years or younger	8	–	8
18–59 years with children older than 5 years	3	2	5
60 years or older	2	2	4
Total interviews	13	4	17

All respondents were visited at their homes and if not available, an appointment was made to return at a suitable time. When respondents refused or were unavailable to take part in the study, another person in the same category was randomly sampled from the database. We over sampled to account for refusals and deaths.

### Recruiting for community member focus group discussions (Focus groups).

In total, six Focus groups were conducted. Respondents for two Focus groups were recruited via convenience sampling, as they belonged to existing community groups (female home-based carers and male community elders) and were stratified by age and gender (Table 2). The recruitment of these community groups was assisted by the voluntary Community Advisory Group (CAG) with permission from the Induna (Village Chief). The concept of the CAG was designed by the Public Engagement Office to ensure that community concerns and ideas are taken fully into account in any research activities that take place in the HDSS site and to provide a forum within the community where research projects are discussed.^46^ This would, we hoped, encourage favorable group dynamics as the respondents knew each other, and it was convenient for us to organize as they met regularly anyway. Recruitment of the four remaining Focus groups was restricted to four villages who had the least amount of HDSS research activity, as advised by the Public Engagement Office, so as not to overburden the community. These respondents were sampled randomly, using the database, from each respective village. A sample of 43 respondents were involved in six focus groups.

**Table 2:** Respondent categories for the antibiotic access and use project Agincourt Health and Demographic Surveillance Systems focus group discussions (Focus groups) and geographical area

Respondent categories	Female respondents	Male respondents	Total
18–29 years old	6 (village A)	7 (village B)	13
30 years and older	7 (village C)	7 (village D)	14
Community group	8 (home-based carers)	8 (community elders)	16
Total Focus groups	23	24	43

#### Field-workers.

Two female Xitsonga-speaking public health field-workers, experienced in qualitative data collection were employed to assist with recruitment and data collection. They were trained by F. W. and a pharmacist regarding 1) antibiotics and use, 2) interview and focus group content, and 3) relevant ethical considerations. When recruiting and interacting with respondents, the field-workers identified themselves as Agincourt HDSS employees conducting research on antibiotics and antibiotics use. They also introduced themselves by explaining which village they resided in (which was not any of the four villages in this research study).

### Data collection.

Data collection was conducted from April to August 2017. Field-workers took turns to lead the focus groups, whilst the other took handwritten field notes to complement the audio recordings. They conducted half of the total number of interviews each. Field-workers went through the study information sheet (Supplemental Appendix 1) in detail with the respondents during the informed consent process, giving them time to ask any questions and seek further clarity where required. Respondents then made the decision whether or not to participate in the study. None of the respondents were known to the field-workers. Light refreshments were provided after each focus group.

Interviews lasted 40–60 minutes and the focus groups lasted around 90 minutes. They were conducted in Xitsonga, the local language. All verbal data were audio-recorded and translated into English while transcribing onto a computer by the same two field-workers who collected the data, within a week after data collection. The field-workers were not involved in interpreting the data for analysis but were approached if anything was unclear in the transcript.

A general interview guide was used across all country sites (site-specific questions will be asked in a further qualitative phase). This was developed by J. K., based on a literature review which identified key issues that could be investigated qualitatively. The questions were then divided up into sections such as 1) accessing care, 2) the suppliers, and 3) the medicines. This process was inductive, but with the concept of access taken as a theoretical construct at the core. The interview guide (Supplemental Appendix 2) focused on the following: any medications taken, where people purchase treatment, and how they pay for it. Several questions were specifically on antibiotic use, where respondents were asked to describe instances when they remembered being prescribed antibiotics and for what condition, and what ABR was. We used the Xitsonga word, *xitsongwatsongwana*, which means “microorganisms,” to refer to bacteria. Our team also coined the term *ku ala ku tira ka tiantibiotic* for “ABR,” which directly translates to “the antibiotic no longer works in my body.”

A study identification number was assigned to each respondent and all identifiers were removed from the data. We have ensured that respondents cannot be identified from any text in quotations. We used different types of qualitative data, each for a specific purpose: the one-to-one interviews allowed for more private settings with people to elicit specific experiences and individual opinions. Whereas the focus groups enabled us to gain a variety of opinions from several different people on the same topic, as they interacted,^49^ also offering insights into community norms. Both sets of data were analyzed in the same manner.

### Data analysis.

Thematic analysis was used to analyze the primary qualitative data to search for themes to organize data in rich detail.^50^ We developed a coding framework directed by categories from the interview guide and dimensions in the Access to Healthcare Framework^37^ that specifies five demand-side abilities associated with healthcare access. Data were coded line by line by J. A. W., with 10 percent recoded by F. W. to check for similar interpretation. When minor discrepancies were found, these were discussed and, if necessary, referred on to J. K. for further input. In reporting this study, we have applied the 32-item Consolidated Criteria for Reporting Qualitative Studies (COREQ) checklist for interviews and focus groups.^50,51^ As per COREQ, we describe the disciplinary backgrounds and research paradigms that our research team collectively share (including experiences in using social theory): health scientist, public health researcher, anthropologist, biologist, demographer, clinician, and statistician. These complementary skills, in combination with our varying degrees of being “insiders” and “outsiders” within the HDSS (as described under “reflexivity”), provided a strong basis for a reflexive and comprehensive set of perspectives during the analysis.

We used NVivo version 10 qualitative data analysis computer software package (QSR International, Doncaster, Australia) to manage our data and look for positive, negative, ambivalent, and nuanced data under each topic. Codes were categorized into themes.^52^ J. A. W., F. W., and J. K. met four times to refine themes until consensus was reached to ensure themes represented the breadth and depth of the dataset.

### Reflexivity.

We were aware throughout the research process of the importance of being reflexive when collecting and interpreting the data. From the onset, we considered where we stood on issues as a means of ensuring that we did not impose our values on the data, thus not taking our own prejudices and values for granted. Given we are a multidisciplinary team, with people from different backgrounds, including some from Agincourt (insiders) and others from elsewhere (outside), this gave us a greater awareness of our respective analytical and disciplinary differences which helped to ensure trustworthiness during the analysis and interpretation stages. The respondents only met the two field-workers and were fully aware of who they were as employees of Agincourt HDSS, by describing who they were and about their role in the study. This was intended to make sure their introductions were transparent as possible as to who they were as well as the intention of the research and data collection process.

### Ethical considerations.

Each field-worker read the consent form out loud to each respondent. Written informed consent and permission to audio-record was voluntarily given before data collection by signature or inked thumbprint, if illiterate. The respondents were given a study information leaflet with the research team’s telephone number in case of withdrawal from the study or to report any ethical concerns.

Ethical approval was granted by the University of the Witwatersrand Medical Human Research Ethics Committee: M160753, the University of Oxford Tropical Research Ethics Committee, OxTREC: 31-15 and the South African Department of Health, Mpumalanga Provincial Health Research Committee: MP_2017RP48_440.

## RESULTS

### Description of respondents.

A total of 60 respondents (34 female and 26 male) were involved in this study (excluding three refusals from the interviews and three dropouts from the focus groups). There were 21 community members unavailable for an interview and 78 community members unavailable for focus groups. Most respondents were unemployed or pensioners, and one community member was a traditional healer. Most people who participated defined themselves as literate and half held the final high school examination of matriculation. As we used the data together during analysis, there was no need to stratify the sample having found no substantive differences in the different groups during our initial readings.

### Themes.

Data are presented under the five themes that comprise the theoretical framework on healthcare access, pertaining to how community members accessed healthcare and treatment within this HDSS. This includes a subsection on community understandings of ABR. There was no substantial difference in the findings from the community members in the interviews and focus groups, and we, therefore, present the data together.

### Ability to seek (personal and social values and autonomy).

The community members have several choices regarding where to seek healthcare. Most of the respondents used the government public health system to access healthcare and treatment. This was for acute illnesses such as bacterial infections requiring antibiotic treatment and for long-term, chronic diseases such as hypertension (medications collected monthly). Although the option to consult a private doctor was available in the locality, low socioeconomic status of the population and social factors resulted in their seeking care predominately from government healthcare providers.

“I think the clinic is the best institution where you can get proper help.” (Interview 1, female, 31 years old, volunteer)

Some people described using traditional healers and then combining biomedical treatments with traditional medicine. Other potential sources of antibiotics included door-to-door sellers, church pastors, or market stall sellers at “pension points” (pop-up monthly village markets on the day pensioners draw social grant state pensions). People have knowledge about the different options of where to seek healthcare from, but patient choice is often guided by financial means, cultural norms, or habit.

### Ability to reach (availability of transport and social support).

Related closely to the ability to seek healthcare is the person’s ability to get themselves or their children physically to the health facility. The cost of public transport, which involves using local minibus taxis from the village to the clinic, was a concern for many respondents. Transport costs were described as an inhibiting factor when a household member required healthcare. Issues around safety were raised by women with young children who had traveled by foot to the clinic. They described how vulnerable and frightened they felt of being attacked and raped.

I was walking back from the clinic with a baby on my back and I was with this other friend of mine. When we were about to cross over on the stream, two men appeared from the bushes and wanted to rape us. We had to run back from where we were coming from with the babies on our backs. And the worst part of it was that we did not have even a single cent [money]. So, we waited for another group of people who were also at the clinic and walked home with them as a group. So, it is a challenge for us when we have to go to the clinic. Those men had knives with them. (Interview 1, female, 31 years old, volunteer)

This illustrates the challenging social setting of this area, as well as some of the concerns that people have to face when traveling to access care.

### Ability to pay (income and social capital).

Most people relied on treatment from primary healthcare facilities, including antibiotics, free at the point of access. However, many described buying supplementary over-the-counter medicines such as painkillers, anthelminthics for deworming, and cough syrups from “spaza” shops (informal convenience shops). They perceived these as affordable and meant they did not have to queue at the clinic. Where possible, people also made home remedies for coughs and diarrhea, using traditional recipes from local plants and trees. Only two of the community members reported consulting at a private doctor and paying for the antibiotic prescriptions at the pharmacy. They did value pharmaceutical medicines as higher quality because they had paid for it, as compared with medication prescribed at the clinic, which was free and so considered lesser quality.

The treatment is for free but people are doubting to use it. Instead they want something that they will pay for. (Interview 2, male, 64 years old, pensioner)

### Ability to engage (information, adherence, and caregiver support).

There was a very mixed understanding of the term “antibiotics.” For the most part, the community had not heard of this word before. However, when described using relevant Xitsonga words (as described in the Methods section), most could then relate to the term. One retired teacher explained what he thought the role of antibiotics was:

According to my understanding, as I was a teacher… when they talk about ‘biotic’, I think they are talking about bacteria. When they talk about ‘anti’, it means they are talking about things that are fighting against bacteria. (Interview 2, male, 64 years old, pensioner)

Some people recalled taking legitimately prescribed and dispensed antibiotics by primary healthcare clinic or by the district hospital.

I am not sure if they fall under antibiotics, but I have once used the Penicillin I was given by the nurse at the clinic. (Interview 3, female, 44 years old, domestic worker)

A variety of bacterial infections, such as tonsillitis, urinary and respiratory tract infections, and wounds, and what antibiotics were used to treat them were described.

Mmm… . I think it [antibiotics] should be taken when you have bladder infection. (Interview 5, female, 47 years old, unemployed)

A few people explained how they needed antibiotics when their “body soldiers” were down, referring to their immune system, a concept that many people have learned in relation to cluster of differentiation 4 count (measurement of white blood cells). Most respondents recognized how to take antibiotics as typically recommended: by finishing the prescribed course, as directed by the nurse or pharmacist.

I must not stop using them just because I am feeling better, I have to continue until they are finished. (Interview 3, female, 44 years old, domestic worker)

Nevertheless, some said they stop taking these tablets, which they knew was ill-advised:

When I feel better, I stop. (Focus group 3, female, below 30 years).

We stop taking the treatment before we even finish the course. We cheat the healthcare workers because we know that they don’t see us and no one will tell them that I did not finish the course of my treatment. (Focus group 4, home-based carer, female)

People who admitted to not finishing the prescribed course of antibiotics said they disposed of unused antibiotics into their outdoor pit latrines. Others said they kept them for the next episode of household illness or to be shared with other family members or neighbors.

We keep them so that we can use them again when we get ill. (Focus group 4, home-based carer, female)

#### Accessing antibiotics from private healthcare.

Community members knew it was not permitted to buy antibiotics directly from the pharmacy without a prescription because of regulation.

Here in South Africa, you cannot get antibiotics without the referral from the doctor. He will prescribe that you have to go and buy in the chemist but besides that, they cannot give them to you. (Focus group 1, female, older than 30 years)

One woman believed that pharmacies would sell antibiotics to her on the basis they are money-making businesses.

Chemists, they don’t care. You can buy without those letters [prescription]. (Interview 6, female, 40 years old, traditional healer)

However, another respondent described how a pharmacy refused to sell antibiotics to her without prescription and told her to go back to the doctor.

I went to the chemist. I didn’t know that I was supposed to come with a letter from the doctor. It’s because those pills were strong… They strictly sent me back and say they want referral letter from the doctor. (Focus group 1, female, older than 30 years old)

Our data provide no account of informal or unqualified vendors selling antibiotics to the community illegally.

Those who had consulted at a private surgery in the past said the doctor had only prescribed antibiotics when necessary.

They [the doctors] know the danger of abusing them. (Interview 6, female, 40 years old, traditional healer)

### Ability to perceive (trust and expectations, health beliefs, and health literacy).

#### Trust and expectations.

Older people, in particular, who were able to afford to buy drugs privately, had purchased tablets from market stall sellers using their pension. Respondents were unsure whether the medication bought was antibiotics as they were sold under the guise of nutritional supplements or analgesics.

They are selling mixed pills which are seven different colors in one sachet. There were saying they are for flu…, others were vitamins. But in one sachet! Others were for pains. People used to buy those pills. That is why they don’t get well as they don’t want to go to the clinic. (Focus group 1, female, older than 30 years)

The young men were conscious that their grandmothers are exploited or “hood winked” at the pension markets. Older women trusted market stall sellers over the clinics and pharmacies and also so that they could avoid queuing.

Our grannies are buying medicines in the pensions. The problem is that they are trusting the medicines they meet on the way than the ones from the chemists or from the clinic ones. (Focus group 2, male, younger than 30 years)

One woman who had bought medicine from the market said it did not cure her ailments:

They are selling in the pension. We are buying as they are advertising them. But after completing the bottles you won’t see any change. (Focus group 1, female, older than 30 years)

This is causing lack of trust among who these sellers are, where they come from, and what the medication is.

#### Health beliefs.

Discussion around health beliefs was not specifically raised by respondents. No one was hesitant to take biomedical medicine owing to their traditional beliefs. Many people described when they seek traditional healers for certain ailments.

As Africans, we have too many different beliefs. There are still people who are consulting to traditional healers. Eh… they are consulting to the hospital when their illness become worse. Their traditional healers by then will say they are not seeing anything wrong with the patient as they will tell you that they tried everything according to their medicine. (Focus group 1, female, older than 30 years old)

The traditional healers are finding that people are turning to biomedical medicine over traditional practices:

As traditional healers, we are not able to work as people are going to buy those medicines straight from the chemist. (Interview 6, female, 40 years old, traditional healer)

Other health beliefs also included prayer.

I understand that demons are operating nowadays. If you don’t pray for your child and think that it’s just a minor headache, you might lose her/him due to death. (Interview 1, male, 23 years old, unemployed)

#### Health literacy.

In general, when primary healthcare nurses dispense antibiotics, the patient receives them in a packet with the drug name printed on the packaging. This does not mean that patients have necessarily understood they are being prescribed drugs classified as “antibiotics.” All respondents reported receiving verbal and written instructions from the nurses on how to take the medication, but they also described how they do not always read or look at the pictorial labels.

We don’t bother to know the names of the medicines and pills that we get from the clinics. And sometimes we don’t even read the name on the containers. (Interview 10, male, 23 years old, unemployed)

Community members do not typically ask for a particular antibiotic at the clinic or pharmacy. With the exception, one person said she specifically requested “amoxicillin” again because it had previously worked. We do not know if she wanted it for the same condition or for another illness.

Prescribed antibiotics were referred to as “so strong” and therefore important to take because from experience, they were effective.

Another thing is that many people are ill nowadays. They don’t know where to go and what to do. That is why they are trusting every information. If you can come with those antibiotics you are talking with, people can buy them very fast. (Focus group 1, female, older than 30 years old)

Community members generally had a limited understanding of the purpose of antibiotics, with some associating them with viral treatment. One woman did not understand why the antibiotic treatment was not reaching her “nerves.”

They will stop the virus to multiply… Antibiotics we are taking are not working. They are saying this virus is hiding in our nerves. (Interview 7, female, 44 years old, housewife)

However, many people had some understanding of antibiotics through their direct or indirect experience of pulmonary TB and cotrimoxazol for HIV/AIDS infection. Treatment of TB, and the prevention and treatment of opportunistic HIV-related infections, which involves taking a long-term course of antibiotics, have both become very much a part of the community’s lives, significantly enhancing people’s general health literacy.

We found that peoples’ frame of reference around antibiotics usually came from their experience of the ongoing TB epidemic, prevalent in this locality. Many respondents knew someone who had been on a 6-month course of combined antibiotics for active pulmonary TB. This direct or indirect experience created a knowledge base that provided people with some understanding of antibiotics. This was relevant to the idea of needing to finish the course and the consequences of not doing so. By applying this knowledge of adhering to antibiotic treatment, many people were able to describe what they thought antibiotics were.

I have heard that once you start using the treatment, you have to finish the course. For instance, when you are initiated on TB treatment, there is a period that they give to take those pills for; and after that period, it is then that they tell you to stop using them. (Focus group 4, home-based carer, female)

Those with personal experience of being diagnosed with TB explained what they had learnt from previously taking antibiotics.

By the time I was diagnosed with TB, I was weak and I was confused. But after taking antibiotics I gained energy and I became strong. I was able to do my household activities. I was told to take them for 6-months (Laughing) and I was not to stop. If I stopped without finishing the course, this can lead the TB not to get cured. I was told that they are working to help me and also to help those who are at home, for the TB not to spread to them. (Interview 7, female, 44 years, housewife)

People had been taught to take their treatment “well.” This was another example of understanding antibiotic knowledge stemming from TB experience.

With those who are on TB treatment, they are feeling good as long as they take their medication in a good way. That’s why they have to complete 6 months while on treatment. This will prevent the spread of TB to other people. (Interview 8, female, 27 years old, unemployed)

#### Antibiotic resistance.

The term “ABR” was unfamiliar to all respondents.

I have never heard of antibiotic resistance. (Interview 9, female, 31 years old, volunteer)

However, as described earlier, people did understand the concept, in relation to TB and HIV/AIDS treatment, and several respondents could give accurate descriptions of what it is.

… In my understanding, there is resistance because people don’t finish the course. And when you use them [antibiotics] again, maybe the illness is getting worse; they will not respond because the first course was not finished and the infections have gotten worse in your system. So the course needs to be finished. (Interview 4, female, 31 years old, unemployed)

A few people gave reference to how the body fights bacteria and how resistance to antibiotics is likely to occur.

The reason why they resist may be because the bacteria have built up and multiplied in a way that the pill cannot be able to treat it. (Interview 5, female, 47 years old, unemployed)

## DISCUSSION

This article provides insights into the ways that people living in a rural South African HDSS site access and use antibiotics, and also their understandings of the concept of ABR. We considered each of the five abilities in the Access to Healthcare framework with respect to where people are accessing and sourcing their healthcare to obtain antibiotic treatment within this rural area. These comprised the abilities to *seek*, *reach*, *pay*, *engage*, and *perceive*.

With regard to peoples’ ability to *seek* healthcare, we found that, as in other South African studies, popular over-the-counter low-cost nonantibiotic medication for fever or coughing in children was commonly purchased,^53^ whereas antibiotics were prescribed primarily by nurses at clinics. To some extent, people in the Agincourt HDSS have autonomy to choose where to get treated (e.g., traditional healer, church pastor, or at the clinic), but this personal choice is limited when it comes to antibiotics, which, by law, are available exclusively from government clinics and from (a few) private doctors. We found that antibiotics are a normalized and an acceptable line of treatment sought, as biomedicine has been part of daily life for some time alongside traditional medicine.

People’s ability to *reach* healthcare services and treatment was subject to the availability of transport to reach the respective facility, and the associated costs, which in some cases meant that antibiotic access was limited. The threat of rape, although only described by one female community member, could mean access can also be compounded by serious safety concerns for females, in particular. This type of social disorganization is not uncommon in South Africa.^54^ We did not find any evidence for people self-medicating antibiotics, largely because the purchasing of antibiotics without prescription was not seen as feasible for the aforementioned reasons.

The actual source and location of where to seek and obtain healthcare corresponded with community members’ ability to *pay* for healthcare, based on their income. Antibiotic medication is free from primary healthcare services, making it the most common derived source. Many people were willing to purchase affordable and commonly available medication, such as paracetamol, used for pain and fever, as found in other South African studies.^54^ For a minority, this “choice” extended to more expensive prescribed medication from a private doctor and then purchased from a local, legitimate private pharmacy. Other studies have found rural patients may become dissatisfied with the clinic and choose to visit a private doctor expecting to get the (right) treatment.^55,56^ Some people in our study also chose to buy medications using their pension money, from alternative unlicensed providers, usually to supplement medication from the clinic. Older people were found to be more susceptible to purchasing from market stall sellers. Little is known about what medicine they are selling, as most published studies are on traditional medication practices.^57,58^ In our data, the illegal selling or buying of antibiotics in this area was not reported. This indicates that at worst, this is only a minor problem in this rural setting, given the regulation of sales of antibiotics. This is unusual in an LMIC setting, as it is well documented that nonprescription sales and the dispensing of antibiotics in more urban areas of Zambia and Tanzania are a widespread problem due to weaker regulatory enforcement.^59,60^

Medication from the clinic was sometimes perceived of lesser quality than the pharmacy’s supplies. Free medication from the healthcare facilities was valued less by several people. The reasoning was that if they were given something for free, it must be of poorer quality than something they paid for. In line with other literature, a study in China has also demonstrated that a “concern with the quality of medicines led to distrust in the public sector” with people preferring medicines purchased from private sector pharmacies.^61^ Yet, we found that people mostly trusted the free antibiotics from the clinic nurses. Factors for this include (potential) trust in the public healthcare system and not always having the financial means to pay for private healthcare.

The community members described their *engagement* with information around antibiotic instructions given by the nurses as satisfactory. However, they did describe instances of not finishing the antibiotic course and disposing of them inappropriately. Some people could not determine the difference between antibiotics for bacterial infections and analgesics for pain relief, perceiving both as “just tablets to get better.” The respondents displayed some understanding of ABR by describing its causes. People were also aware that antibiotics are strong, with the capacity to cure a range of ailments.^62^ Even though people knew they should finish a course of antibiotics, some conceded that they do not always follow the verbal or written instructions. This was also found in a study by Friend-du Preez et al.^54^ on health seeking behavior for childhood illness in urban South Africa, whereby antibiotics were not always used as intended or according to the recommended instructions.^63^

The community’s ability to understand antibiotics and ABR was derived from their experiences of other well-known illnesses, such as TB, and opportunistic infections, such as pneumonia, often associated with HIV/AIDS. The relationship between new concepts and existing notions of medicine and care in the context of TB and HIV/AIDS is of interest. These diseases have played a prominent role in the community over a long period of time and have been covered extensively by public health campaigns. Therefore, we found that people’s health literacy, their beliefs, trust and expectations (their ability to perceive), and their level of health information, adherence, and empowerment (their ability to engage) were often based on illnesses such as TB. Through this knowledge, people could grasp the concepts of “antibiotics” and “resistance” even if they were not familiar with the words themselves. It is not yet clear whether by “grasping” new concepts of “antibiotics” and “resistance,” the respondents’ concepts overlapped with clinical concepts. For example, it could be possible that people are still not able to distinguish antibiotics from other medicines, and that, therefore, the introduction of new concepts alters behaviors for other medicines as well—with potentially unforeseen consequences. Much research has been conducted on social and psychological factors influencing health outcomes,^64,65^ and this has had a direct impact on the ways in which both TB and HIV/AIDS have been addressed in South Africa. Media campaigns targeting rural and urban communities have successfully sought to convey information about the two diseases,^66,67^ which suggest that a similar method could be used in the fight against ABR. Community groups could also be established to discuss antibiotic stewardship and the impact of resistance, following principles developed in an urban township in Cape Town, in which patient adherence groups were found to be effective as a model of care.^68^

These results suggest that people are aware of antibiotics in some capacity and have specific local interpretations of this type of medicine. Also, people can relate to the concept and its extension to ABR because of their TB/HIV knowledge. We do not know the extent to which these new conceptions were specific to antibiotics rather than just gaining new knowledge about medicine more generally. Further research would have to discover whether these new conceptions mapped perfectly or imperfectly onto clinical definitions as these existing data do not give light to this.

### Study strengths and limitations.

As random sampling for the interviews were used, this study included respondents from four different HDSS villages. This was considered a strength because it meant their individual healthcare experiences differed and so were the clinics that healthcare was accessed from. By using community leaders to assist with focus group recruitment, we were able to recruit preexisting groups of people, already known to one another. Although this may have encouraged a flowing discussion, however, we recognize that the community leaders may have selected people for their own particular reasons, and this could have introduced some bias into the dataset.

A further limitation of this study was the difficulty in investigating a topic that the HDSS population were unfamiliar with when asked about medical terms that cannot be directly translated into the local language. To counteract this problem, we used Xitsonga words *xitsongwatsongwana* (microorganisms/bacteria) and *ku ala ku tira ka tiantibiotic* (ABR). In the end, we found issues around terminology did not in fact matter because although people did not know the “antibiotic,” they could mostly understand the concept.

For three of the five themes presented in the conceptual Access to Healthcare Framework, significantly more data were found (for the abilities to pay, to engage, and to perceive). The abilities to seek and to reach antibiotics could therefore be investigated further in future research into this topic. Also, we used a different theoretical framework to help analyze and interpret our qualitative data compared with the overall mixed methods design of the larger, six-country study (Sen’s Capability Approach^36^). Sen’s theory did not fit with the specific research questions that we were trying to answer here. Furthermore, the data collection methods that we used allowed us to collect a standardized set of qualitative data from all six participating countries, which in turn will permit comparisons between all of the sites. Although there was the opportunity for probing specific topics that arose during the semi-structured interviews and focus groups, and we sought to encourage an open atmosphere during the discussions, this need for standardization required us to follow a broad set of predefined topics. It was not feasible, given the nature of the larger ABACUS project, to engage in more ethnographic research which could have opened up more unexpected lines of enquiry, concerning, for example, different conceptions of antibiotics and the meanings behind existing behaviors.

The field-workers who collected, translated, and transcribed the interviews and focus groups were not involved in the analysis and interpretation of the data. Conversations between the two field-workers and researchers post-interviews allowed them to give feedback about the provisional findings. Not specific to our study, further limitations include the positionality and reflexivity of us, as the authors, and how we acted on our pre-assumptions and consideration into how we influenced the research process,^69^ and also the relationship between the local field-workers and respondents (both of whom are from the HDSS population).

### Implications for policy, practice, and future research.

Many of our respondents expressed a desire to learn more about antibiotics as a result of participating in the research. This finding is not uncommon: a study by Norris^70^ found that a Samoan population in New Zealand also lacked understanding around the antibiotics and wanted to know more about biomedical medicine. The study concluded that developing appropriate messages around preventing and managing infections and “building on culturally based practices is a safe strategy.”

Two sources of information already available to the community are the nurses’ health talks at clinics and communications from community healthcare workers or home-based carers, whose role includes teaching and advising the community about how to take medications. These may be valuable avenues for informing people about their role in ABR, both when taking antibiotics themselves or when administering them to their children. By making the nurses and community healthcare workers’ pledge to be “Antibiotic Guardians,”^71,72^ this could be part of the South African Department of Health wider antibiotic stewardship programme.^6,73^ Both groups of healthcare workers can be trained to include relevant patient information about safe antibiotic use, tailored to the local communities’ needs and existing knowledge levels and paradigms. This would speak to “Education and Communication and Public Awareness,” one of the pillars (objectives) of the “South African Antimicrobial Resistance Strategy Framework”^74,75^ (which also include enhancing ABR surveillance, governance, stewardship, and prevention).

Antibiotic awareness education could also be supported by national public health campaigns via social media and standard communication techniques. This approach is being used in the United Kingdom, for example, where the National Health Service has launched a campaign called “Treating your infection without antibiotics,”^76^ using principles similar to those used in the “World Antibiotics Awareness Week” campaign.^77^ There are potential limitations of knowledge and awareness campaigns that need to be evaluated,^78^ and other complementary activities—within and outside of the public health sector that might be necessary to assist in altering behavior. Broader structural interventions are necessary; yet in this area, it has not yet become immediately clear what these could be as the antibiotics are not available illegally outside the health system, so there are no laws that need to be enforced better there, which is usually a target for intervention (as is the case for the other ABACUS countries). In that sense, awareness raising is one of a very limited number of options open to the Department of Health in this setting (and we are aware of the inherent limitations even these have).

Many policy debates have been focused on the right of access to antibiotics,^79^ rather than on patient education and safety. This study gives evidence for the need for more education and training around taking antibiotics appropriately for the community and the healthcare workers. However, by improving patient education around antibiotics and resistance will not solve unsafe antibiotic use among patients as this is unrealistic. Also, a focus on education and knowledge can ignore structural and contextual facets of behavior, for example, cultural meanings of good care, economic constraints, discrimination, patients’ despair and experiences of uncertainty, and social relationships between patients and healthcare workers. If substantial changes to a country’s structures are not addressed, then bringing about safer antibiotic use may not happen. We are not in a position to make substantial changes to the structures and the context, thereby bringing about appropriate antibiotic use, although we may be in a position to bring about an improvement in person’s understanding of the issue through education and awareness-raising programs.

Another source of public health education can be using the community pharmacy model whereby pharmacy staff champion AMR and ABR and are empowered to initiate conversations with patients around antibiotics.^80^ The impact and practicality of trying to “empower” local pharmacists to be AMR/ABR stewards may be problematic. It may possibly entail unforeseen social consequences (e.g., changing the relationships between patients and the health system) and pharmacists may comply strategically, to pursue non-altruistic goals that could entail worsened patterns of pharmaceutical use.

At national or provincial level, an additional intervention could involve the regulation of the drug sales at pension points and to check their drugs are safe, legal, and not counterfeit. Another study to interview the suppliers of medication in the Agincourt HDSS site is presently underway and we may get a better sense through this of the medications they are selling, as well as whether these may include any antibiotic and/or counterfeit drugs.

There has been a modest number of studies on social interventions in first-line public health primary healthcare settings.^73,81^ All of these educational and awareness-raising interventions outlined would need to be developed, implemented, and then evaluated rigorously, before being scaled up.

## CONCLUSION

From the perspective of the Access to Healthcare framework, our study has found that despite free provision at primary healthcare clinics, “seeking” and “reaching” antibiotics could be problematic because of difficulties reaching the facilities. Moreover, “paying” for the transport to the clinics constitutes a significant barrier to many people. The respondents’ ability to “engage” with and “perceive” what antibiotics and ABR are was based largely on their prior health literacy, which, for many people, has been developed in relation to TB and HIV/AIDS treatment. We recognize education is only one facet in addressing the misuse of antibiotics yet factors such as poverty, insecure income, and lack of access to healthcare may impact of the benefit of this education.^82^ If locally contextualized and used in coordination with suitable training for healthcare workers, the safe use of antibiotics could be promoted through the range of preexisting materials on community antibiotic education, from organizations such as the WHO.^78^

The ABACUS project aims to provide an empirical basis for informing future, patient-level social interventions for appropriate and safe ABACUS across six LMICs. This study’s focus on knowledge and social practices related to antibiotic use has implications related to educational and awareness-raising interventions relevant to rural South Africa, rather than social intervention recommendations that focus on the social and cultural environment in which people consider and seek care. Educational campaigns for this specific context could be developed by drawing on people’s experience with TB and HIV/AIDS treatment. By developing effective patient education and health promotion materials to reduce unsafe antibiotic use, we need to understand how people talk about and think about antibiotics and infection.^14,83^ Also, the absence of existing notions of antibiotics and resistance among marginalized groups requires other forms of interventions that involve different approaches than standardized material on community antibiotic education. Rather, a contextually relevant long-term antibiotic-based “curriculum” that is driven by the South African health system, for members of the public to understand the concepts of illness and associated antibiotic treatments, would be welcomed.

## Supplementary Files

Supplemental appendices
