# Supplementary material for: Rural South African Community Perceptions of Antibiotic Access and Use: Qualitative Evidence from a Health and Demographic Surveillance System Site
Source: Am J Trop Med Hyg. 2019 Apr 15;100(6):1378–90. doi: 10.4269/ajtmh.18-0171 (PMC6553901; doi:10.4269/ajtmh.18-0171)
Supplement: Supplementary file 1 [file tpmd180171.SD1.pdf]

The following are supplemental materials and will be published online only

Supplemental Appendix. 1
Participant information sheet

INDEPTH ABACUS study. Community-level antibiotic access and use in low- and middle-income countries; finding targets for social interventions to improve rational antimicrobial use.

Dear sir / madam,

As you may know, researchers from Agincourt HDSS in collaboration with the INDEPTH network have done many important studies in your living area. Now we would like to invite you to take part in our INDEPTH ABACUS study.

Purpose: The aim of this study is to compare the use of medicines between 6 countries in Africa and Asia. It is important that medicines are accessible and properly used by all people living in these countries. The INDEPTH ABACUS study will give us a snapshot of the current situation, and it will also tell us how to improve things in terms of supply and use of medicines in your community.

Invitation: We invite you because you are a representative member of the community that we study. We feel that your experience will be useful in understanding more about the supply and use of medicines in your community.

Method for interviews: If you are interested in joining this study, we will have an individual in-depth interview. The interview is about your experience with obtaining and using medicines. We would like to know more about where medicines are obtained, how they are used, and for what reasons. The interview will take about 60 minutes, and will be audio recorded. If you do not want the interview to be audio recorded, alternatively you may consent with a written report. The interview will be held at a time and place that provides sufficient privacy, and is agreed upon by you and the researcher.

Method for FGD: If you are interested in joining this study, we will have a focus group discussion, together with 5 to 7 fellow community members. The interview is about your experience with obtaining and using medicines. It will take about 90 minutes, and will be audio recorded. The interview will be held at a time and place that provides sufficient privacy, and is agreed upon by you and the researcher.

Risks and benefits: Although the interview could raise practices that may be seen as imperfect, the study staff will not take action on this. The audio recordings will be safely stored and will not be identified with your personal information. Your information will be kept strictly confidential, and will not be shared with people outside the research group. The results of this study will only be available for scientific and public health purposes. You will not be personally named in any publication. There is no risk to your health from being in the study. Although there are no direct benefits for you from being in the study, we anticipate that your input will help to improve the supply and use of medicines in the future.

Basics: You are free to choose if you want to take part in this study. Also, you can withdraw your consent at any time without further explanation, and without any effect on your healthcare. This project has been

reviewed by, and received ethics clearance through, Oxford and Wits. This project is funded by the Wellcome Trust.

Your involvement in the INDEPTH ABACUS study is greatly appreciated. If you are happy to take part in the study, please read and sign the attached consent form.

Thank you,
[Name], local Principal Investigator, [contact details].

If you have a concern about any aspect of this project, please speak to the relevant researcher ([tel. No.]) [or their supervisor ([tel. No.]), who will do his/her best to answer your query. The researcher should acknowledge your concern within 10 working days and give you an indication of how he/she intends to deal with it. If you remain unhappy or wish to make a formal complaint, please contact the chair of the Research Ethics Committee at [title of local university or HDSS] (using the contact details below) who will seek to resolve the matter expeditiously:
[Contact details for the local ethics committee]

Supplemental Appendix. 2
Community member in-depth interview guide

Accessing treatment

1) How would you rate your overall health?
2) What do you do when you have any of these conditions: [Probes: self-medication, health centre, pharmacy, traditional healer etc.]
    a. Fever
    b. Headache
    c. Cough/cold
    d. Diarrhea
    e. Wound or skin infection
    f. Severe illness
    g. A sick child

    Why do you choose these particular healthcare options?
3) How long does it take you to get to the place/s where you usually go for treatment, and how much does it cost to get there (if transport is necessary)? Is this convenient, or would you prefer something easier to reach?
4) How do you cover the costs for treatment when you or a member of your family is unwell? How easy is it for you to cover these costs, and what do you do if you are unable to cover them.

5) What would you do if there is a particular medicine you are seeking but the supplier doesn’t have it? (Probe: buy an alternative medicine from the supplier, go somewhere else etc.)
6) Do you ever ask for or use medicines from other people who have similar illnesses to your own? Details (e.g. from whom, which medicines, for which illness, reasons, etc.)

The supplier/seller of medicines

7) Do you usually ask the supplier for a particular medicine, or do you usually let them decide which would be best for the illness you have?
8) Do you usually receive instructions (verbal or written) for using the medicines from the supplier? In general, do you think they are well informed about the medicines they sell, so that you would trust their word and want to follow their instructions? Details. (Probe: by type of suppliers)
9) Do you ever buy less medicine / fewer pills than recommended by your healthcare provider? *[Probe: do you sometimes not have enough money to buy the full course of treatment?]* Details.

The medicines

10) What sort of medicines, if any, do you have at home at the moment, and what are these medicines for?
11) Do you know what antibiotics are, and what sort of conditions they treat? Can you give any examples of antibiotics that you know about?
12) Have you or anyone in your close family ever used this antibiotic? What condition/illness was it used for? Was it effective or not? Do you have a preference for an antibiotic for certain conditions? If so, based on what? Other details.
13) Do you think that antibiotics have any particular benefits or risks? *[Probes: side effects, antibiotic resistance]*.
14) If you want to buy antibiotics, what do you do? Do you have to get a prescription from the doctor first, or can you just go directly and buy them at the pharmacy/supplier? *[Probe: can you give more details about the type of supplier you go to, or describe a recent encounter with a supplier?]*
15) Only if you are willing to, you may answer the following questions, but no answer is needed. Are any of the medicines that you have at home now antibiotics? If so, what illnesses were they bought as treatment for?
16) Have you ever bought more antibiotics than you needed at the time, for future use? Details.
17) Do you sometimes stop taking your antibiotics before your pills are finished? Details (how and why). *[Probes: side effects, not sick anymore]*.

18) If you ever have unfinished antibiotics, what do you do with them? *[Probes: do you keep them? Do you ever give unused doses to other people who have the same illness you had?]*
19) Are you aware of any special instructions that you need to know about when you take antibiotics? Details.
20) Have you ever heard of antibiotic resistance? Do you know what it is, how it's caused, and what its implications are? Is there anything on this topic that you would like to know more about? Details.
21) Do you think that the medicines you buy are usually of good quality? Have you ever had any bad experiences with medicines because you thought they were not good quality? Details.
22) Do you know whether medicines have an expiry date? If you think so, can / do you usually check the expiry date of the medicines you use? What do you do if the medicines are past their expiry date?
23) In general, where and how do you/the community learn about medicines? If you wanted to know more, where would you go? What would be the best source of information about medicines for you?

Community member focus group discussion guide

Accessing treatment

1) Please describe the different alternatives that people in this community use to receive treatment if they are ill (i.e. health centre, pharmacy, traditional healer, medicine peddler, etc). What are the main challenges people face in accessing these facilities in terms of mode of transport, time, and cost?
2) In general, how would you describe the *quality* of the health services (public and private, including pharmacies and other medicine suppliers – and referring to staff, equipment, *and* medicines) that serve your community? What, if anything, do you think could be improved?
3) How are the costs of healthcare covered by people in this community? Which are the ways of obtaining medicines, and do you have to pay for the medicines yourself? *[Probes: health insurance, charity, out-of-pocket, other?]*

The medicines

4) Do you think that the medicines available for people in this community are usually of good quality? Have you heard of any bad experiences that people have had with medicines that were caused because they were not good quality? Details.
5) Do you know what antibiotics are, and what sort of conditions they treat? Can you give any examples of antibiotics that are available in this community?
6) Do you think that antibiotics have any particular benefits or risks? *[Probes: side effects, antibiotic resistance]*.

7) Under what circumstances do people in this community take antibiotics without a prescription or a without a recommendation from a health care worker?
8) What do you think could be done to ensure the safe use of medicines in this community?
9) Is there any sort of advertising here for medicines, and in particular for antibiotics? If yes, how far do you think this advertising influences people's choices about the medicines they buy?
10) In general, from where do people in this community purchase antibiotics?

The suppliers/seller of medicines

11) Do people usually receive instructions (verbal or written) from the supplier for using the medicines they buy?
12) In general, do you think that suppliers are well informed about the medicines they sell, so that people trust their word and want to follow their instructions? Details. [*Probe specifically for antibiotics*.]
13) Do people ever buy less medicine / fewer pills than recommended by their healthcare provider? IF so, why does this happen? Details. [*Probe specifically for antibiotics*.]

Antibiotic resistance

14) VIGNETTE: A person living in this community had a certain health condition. He went to the hospital and was given some the most appropriate medication for his condition. He took the full course of the medicine but he did not feel better. A few days later he went to another hospital for the same condition and he was given the same medicine that he was given at the first hospital. Once again, he took the full course of the medicine as the doctor instructed and still did not feel better.
    a. What do you call this condition where you take medicines for a condition but the condition does not go away?
    b. Why do you think the medication is not working?
    c. What do you think the person should do?
    d. How do you think the person can be helped?
    e. If someone takes antibiotics for a condition and it does not work, what do you call that condition?
15) What do people do with unfinished doses of antibiotics? Details (how and why).
16) Do people sometimes stop taking their antibiotics before they have finished all the pills they obtained? Details (how and why). *[Probes: do people save them? Do people ever give unused doses to other people who have a similar illness?]*
17) Have you ever heard of antibiotic resistance? Do you know what it is, how it's caused, and what its implications are? [*Question to be adapted according to responses to previous questions, in particular Number 5*.] Is there anything on this topic that you would like to know more about? Details.

18) In general, where and how do people in this community learn about medicines? What do you think would be the best way to inform people in your community about proper antibiotic use and the dangers of antibiotic resistance?
